# A propos d’une tuméfaction sternale

**DOI:** 10.11604/pamj.2017.28.146.13374

**Published:** 2017-10-16

**Authors:** Dhia Kaffel, Hela Kchir

**Affiliations:** 1 Rheumatology Department, Kassab Institute, Manouba, Tunisia; 2 Gastroenterology B Department, Rabta Hospital, Tunisia

**Keywords:** Nétastase, carcinome hépatocellulaire, hépatite virale C, cirrhose, paroi thoracique antérieure, Metastasis, hepatocellular carcinoma, hepatitis C virus, cirrhosis, anterior chest wall

## Abstract

We here report the case of a 64-year old woman followed up for cirrhosis due to hepatitis C virus who didn't respond favorably to antiviral treatment. During her last follow-up visit, she reported the occurrence of painful anterior chest hump. Physical examination showed hard immobile mass at the level of the manubriosternal joint. The patient underwent sternoclavicular CT scan, which objectified expansive osteolytic lesion centered upon the sternal manubrium invading the soft tissues (A, B). The biopsy revealed malignant papillary epithelial tumor expressing pancytokeratin and CK7. Patient's profile suggested the presence of a metastasis from cholangiocarcinoma or osteophilic tumor. Gynecological examination, associated with mammogram and breast ultrasound, excluded a gynecological origin. The diagnosis of thyroid tumor was excluded on ultrasound. Chest CT scan showed multiple secondary pulmonary nodules. Abdominal angioscanner revealed the presence of a tissutal hepatic mass measuring 6 cm invading the portal bifurcation with portal vein thrombosis, suggesting hepatocellular carcinoma (HCC) (C). Given the discrepancy between anatomopathological data and morphological data, immunohistochemical study of the anti-Hep-Par-1 was performed, showing antibody expression on tumor cells. The diagnosis of manubriosternal metastasis from poorly differentiated hepatocellular carcinoma was retained. The patient was recommended to undergo chemotherapy. Bone metastases revealing HCC are exceptional. However, HCC should be suspected in patients with lytic bone lesion, especially in patients with chronic liver disease. Given its poor prognosis, treatment is based on palliative therapy with the aim of improving mainly the quality of life of patients.

## Image en médecine

Nous rapportons le cas d'une femme âgée de 64 ans suivie pour hépatite virale C au stade de cirrhose ayant échoué au traitement antiviral. Lors de sa dernière consultation, la patiente a signalé l'apparition d'une voussure douloureuse thoracique antérieure. L'examen physique a révélé une masse dure et immobile en regard de l'articulation manubrio-sternale. Une tomodensitométrie (TDM) sterno-claviculaire a été réalisée, objectivant une lésion expansive ostéolytique le manubrium sternal envahissant les parties molles (A, B) et dont la biopsie a révélé une tumeur maligne d'allure épithéliale d'architecture papillaire exprimant la pancytokératine et la CK7. Ce profil était évocateur d'une métastase d'un cholangiocarcinome ou d'un cancer ostéophile. Un examen gynécologique spécialisé avec une mammographie et une échographie mammaire ont éliminé une origine gynécologique. Une échographie cervicale a écarté une tumeur thyroïdienne. La TDM thoracique a révélé de multiples nodules pulmonaires d'allure secondaire. Un angioscanner abdominal a montré la présence d'une masse tissulaire hépatique de 6 cm envahissant la bifurcation portale avec thrombose porte évocatrice d'un carcinome hépatocellulaire (CHC) (C). Devant la entre les données anatomopathologiques et morphologiques, une étude immunohistochimique par l'anti-Hep-Par-1 a été réalisée montrant une expression de cet anticorps par les cellules tumorales. Le diagnostic de métastase manubriosternale d'un carcinome hépatocellulaire peu différencié a été retenu. La patiente a été proposée pour chimiothérapie. Les métastases osseuses révélant un CHC sont exceptionnelles. Toutefois, il faut les évoquer devant toute lésion osseuse lytique, surtout chez les patients atteints d'hépatopathie chronique. Vu leur pronostic sombre, leur traitement est palliatif, visant essentiellement à améliorer la qualité de vie des patients.

**Figure 1:**
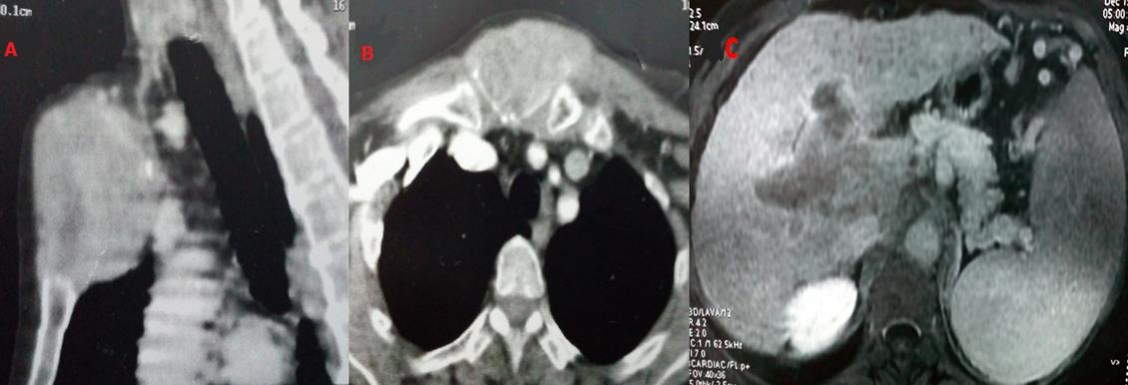
(A,B) TDM de la paroi thoracique antérieure montrant une lésion expansive ostéolytique centrée sur le manubrium sternal avec un envahissement des parties molles; (C) angioTDM montrant une masse tissulaire hépatique envahissant la bifurcation portale avec thrombose porte

